# Syphilitic Panuveitis and Rhegmatogenous Retinal Detachment: Diagnostic Pitfalls and Treatment Considerations

**DOI:** 10.3390/medicina62040798

**Published:** 2026-04-21

**Authors:** Sofija Davidović Terzić, Siniša Babović, Svetlana Pavin, Aleksandar Miljković, Nikola Denda, Sava Barišić

**Affiliations:** 1 Faculty of Medicine, University of Novi Sad, 21000 Novi Sad, Serbia; aleksandar.miljkovic@mf.uns.ac.rs (A.M.); 902018d24@mf.uns.ac.rs (N.D.); 2 Clinic for Eye Diseases, University Clinical Centre of Vojvodina, 21000 Novi Sad, Serbia; sinisa.babovic@kcv.rs (S.B.); svetlanapavin@gmail.com (S.P.); savabarisic@gmail.com (S.B.)

**Keywords:** ocular syphilis, panuveitis, posterior uveitis, retinal detachment, vitreoretinal interface, masquerade syndrome

## Abstract

Syphilitic panuveitis is a severe and diagnostically highly challenging manifestation of ocular syphilis. Its predominant posterior-segment involvement and its tendency to mimic noninfectious or viral uveitis may delay etiologic recognition and increase the risk of permanent vision loss. Rhegmatogenous retinal detachment (RRD) is a rare but vision-threatening complication that likely reflects advanced, inflammation-induced disruption of the vitreoretinal interface. A narrative literature review was conducted using the PubMed, Scopus, and Web of Science databases (January 2000 to 10 September 2025). Studies addressing the clinical presentation, imaging findings, pathophysiology, and management of syphilitic panuveitis and associated rhegmatogenous retinal detachment were analyzed. Infectious mimickers were also presented, with particular emphasis on West Nile virus (WNV). Evidence was synthesized qualitatively. Posterior uveitis and panuveitis are one of the most common ocular manifestations of syphilis. Posterior segment involvement in ocular syphilis is frequently bilateral, typically presenting with dense vitritis, retinal vasculitis, and optic neuropathy. RRD is a rare presenting complication, most often developing in areas of prior inflammatory retinitis and arising due to retinal necrosis, persistent vitreoretinal traction, and early proliferative vitreoretinopathy, which increases surgical complexity and may limit functional recovery. HIV coinfection often modifies disease severity. In relevant endemic or seasonal settings, WNV-associated ocular inflammation represents an important diagnostic pitfall. Syphilitic panuveitis should be considered early in patients presenting with unexplained posterior uveitis or panuveitis. Routine testing for syphilis and HIV in the uveitic laboratory palette, together with targeted evaluation for infectious mimickers, is essential to reduce diagnostic delay and avoid inappropriate immunosuppression. RRD should be recognized as a marker of advanced, inflammation-induced vitreoretinal interface damage requiring timely antimicrobial therapy and early involvement of vitreoretinal surgery.

## 1. Introduction

Panuveitis represents a severe form of intraocular inflammation involving the anterior chamber, vitreous, retina, and choroid, and it can arise from a broad spectrum of infectious and noninfectious etiologies [1]. Infectious causes are of particular clinical importance because of their potential for rapid progression, diagnostic uncertainty, and permanent vision loss if not recognized and treated promptly. Syphilis is notable for its ability to mimic a wide range of uveitic phenotypes and has therefore been named as the “great imitator” [2,3,4]. Recent increases in syphilis incidence in general have been accompanied by more frequent reports of its ocular involvement [5]. Ocular syphilis is considered to be a form of neurosyphilis, and around 20% of patients with ocular manifestations usually have associated syphilis-related neurological abnormalities. Syphilitic eye involvement may occur at any stage of disease and sometimes can even be the presenting manifestation, which contributes to delayed diagnosis in routine ophthalmology practice. Posterior uveitis and panuveitis are among the most common clinical presentations, apart from isolated anterior uveitis, and are frequently bilateral, often characterized by dense vitritis, retinal vasculitis, and optic nerve involvement, with poorer visual outcomes when treatment is delayed [6,7]. Rhegmatogenous retinal detachment is a rare but severe complication of syphilitic panuveitis and likely reflects advanced inflammation-induced damage at the vitreoretinal interface [8]. Its incidence and underlying mechanisms are not fully defined, but proposed contributors include retinal necrosis and thinning, persistent inflammation with chronic vitreoretinal traction, and subsequent retinal breaks [9,10,11,12]. The diagnosis of severe panuveitis is further complicated by overlapping phenotypes across infectious uveitis cases, which may necessitate an expanded, infection-oriented diagnostic approach. West Nile virus, although less common, can mimic ocular syphilis and represents a clinically relevant diagnostic pitfall in appropriate seasonal or endemic settings [13,14,15,16,17].

Although previous reviews have addressed ocular syphilis and posterior syphilitic uveitis, the interplay between syphilitic panuveitis, rhegmatogenous retinal detachment, and infection-oriented diagnostic decision making remains insufficiently explored. This review provides a clinically focused synthesis of these interrelated issues, with particular emphasis on the pathophysiologic and surgical relevance of retinal detachment, key diagnostic pitfalls including infectious mimickers such as West Nile virus, and the implications for earlier recognition and treatment sequencing.

## 2. Materials and Methods

### 2.1. Scope of the Review

This narrative review summarizes the available clinical evidence on syphilitic panuveitis, with a specific focus on areas that remain insufficiently integrated in previous studies: the pathophysiologic basis and clinical significance of rhegmatogenous retinal detachment, the diagnostic implications of overlapping infectious phenotypes, and the consequences of delayed etiologic recognition for treatment sequencing and visual outcomes.

### 2.2. Data Sources and Timeframe

The literature search was conducted in PubMed, Scopus, and Web of Science, covering publications from 1 January 2000 to 10 September 2025. Only English-language publications were considered. Additional relevant references were identified by manual screening of the reference lists of included articles.

### 2.3. Search Strategy

The search strategy combined controlled vocabulary (MeSH terms) and free-text keywords. Search terms included combinations of syphilis, ocular syphilis, syphilitic uveitis, panuveitis, posterior uveitis, vitritis, retinitis, chorioretinitis, retinal vasculitis, optic neuritis/neuroretinitis, rhegmatogenous retinal detachment, retinal break, proliferative vitreoretinopathy, vitreoretinal traction, and vitrectomy, as well as terms related to infectious mimickers (e.g., West Nile virus, WNV, viral chorioretinitis, and infectious uveitis mimickers).

### 2.4. Eligibility Criteria

Eligible publications included studies reporting on: (1) the clinical presentation of syphilitic panuveitis/posterior uveitis; (2) the diagnostic work-up and differential diagnoses (serology, cerebrospinal fluid assessment when relevant, and multimodal imaging); (3) imaging findings (fundus photography, fluorescein angiography/ICGA when available, OCT, ultra-widefield imaging, and ultrasonography); and/or (4) therapeutic approaches and outcomes, including the surgical treatment of vitreoretinal complications. Prospective and retrospective clinical studies, observational cohorts, and case series were included, along with selected narrative or systematic reviews that contributed contextual or integrative information. Studies without a clear clinical correlation with syphilitic uveitis or with insufficient data on phenotype and/or outcomes were excluded.

### 2.5. Handling of Case Reports

Case reports were not used as the primary basis for inference but were included selectively when they provided clinically meaningful insights into rare or diagnostically challenging scenarios (e.g., bilateral rhegmatogenous retinal detachment, well-documented coinfection, or atypical phenotypes mimicking non-infectious uveitis or viral retinitis).

### 2.6. Study Selection and Data Extraction

Two authors independently performed the initial screening of titles and abstracts, followed by full-text evaluation of studies meeting the eligibility criteria; any disagreements were resolved by consensus. The following variables were extracted from the included studies: study design, population characteristics (including HIV status when reported), predominant clinical presentation, key imaging findings, therapeutic approach (antimicrobial and anti-inflammatory therapy), and vitreoretinal complications and outcomes (anatomical and functional, when available).

### 2.7. Evidence Synthesis and Quality Considerations

Given the substantial heterogeneity in study design, patient population, and outcome reporting, the evidence was synthesized qualitatively. A formal risk-of-bias assessment was not performed because the aim of this review was to integrate clinical and pathophysiological insights and to highlight diagnostic pitfalls rather than to provide a comparative effectiveness assessment. Findings were organized thematically around: (1) predominant posterior-segment phenotypes; (2) the diagnostic algorithm and key pitfalls; (3) the vitreoretinal mechanisms of complications, with a focus on rhegmatogenous retinal detachment; and (4) treatment principles and therapeutic sequencing.

### 2.8. Conceptual Framework

**Figure 1:**
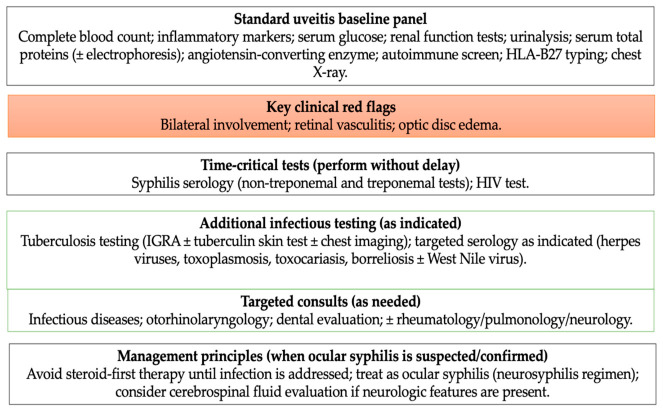
Diagnostic approach to severe posterior uveitis or panuveitis with suspected infectious etiology. The scheme highlights the clinical red flags prompting an infection-oriented workup, recommended initial testing (syphilis serology and HIV), escalation to targeted investigations based on clinical and epidemiologic context (e.g., West Nile virus, herpes viruses, tuberculosis), and key management principles, including avoidance of corticosteroids as first-line therapy and prompt neurosyphilis-directed antimicrobial treatment when indicated.

The diagnostic framework derived from the gathered evidence is presented schematically (Figure 1).

### 2.9. Clinical Images and Data Privacy

Two representative clinical images were included. They were obtained from the routine clinical practice database at the Clinic for Eye Diseases, University Clinical Centre of Vojvodina, Novi Sad, Serbia, solely for illustrative/educational purposes, to complement the narrative synthesis. All images were fully de-identified prior to inclusion (removal of patient names/initials, medical record numbers, dates, and any other identifiers) and were not linked to any additional patient-level dataset. Written informed consent for publication of the images was obtained from the patient(s).

## 3. Clinical Spectrum and Evidence Synthesis

### 3.1. Ocular Syphilis and Panuveitis

Syphilis is a bacterial infection caused by Treponema pallidum and continues to be a significant, resurgent global public health problem. The recent rise in systemic syphilis has been paralleled by an increase in reported cases with ocular involvement [4]. Ocular syphilis can manifest at any stage of infection; it is not uncommon for it to be the first or even the only clinical manifestation of the disease. For this reason, during the routine diagnostic workup of a uveitis-presenting patient, this etiology often remains unrecognized in the early stages [1,2,3]. Data from modern review articles and cohort studies show that the posterior segment of the eye is most commonly affected in ocular syphilis, along with the anterior uveitis. Panuveitis and posterior uveitis are described as the dominant clinical forms, which often occur bilaterally and are characterized by an aggressive course of the disease. These clinical presentations are associated with severe visual impairment, especially when the diagnosis and initiation of adequate antimicrobial therapy are delayed [5,6,7]. In support of the severity of the clinical picture, recent clinical series and retrospective analyses indicate the frequent occurrence of dense vitritis, posterior segment inflammation, retinal vasculitis, and optic nerve involvement in patients with syphilitic uveitis (as a form of neurosyphilis) [18]. These findings clearly indicate that the occurrence of severe posterior uveitis or panuveitis, especially when associated with retinal vasculitis or optic nerve papilloedema, should prompt urgent serological testing for syphilis, regardless of the patient’s anamnesis and estimated demographic or behavioral risk [19]. One of the essential features of ocular syphilis, which is consistently emphasized in the literature, is its broad clinical spectrum. Precisely for this reason, it can largely mimic autoimmune forms of uveitis or viral retinitis, which further complicates the initial differential diagnosis and increases the risk of late establishment of an accurate diagnosis [20]. To contextualize the clinical spectrum and diagnostic implications of ocular syphilis, representative contemporary studies and guideline-based sources, together with selected quantitative findings where available, are summarized in Table 1.

Ocular syphilis is most commonly associated with a variety of inflammatory manifestations, which may include nongranulomatous anterior uveitis, severe vitritis, chorioretinitis or retinitis, retinal vasculitis, and optic disc edema or neuroretinitis. Serous or exudative retinal detachment is less common but is clinically significant when present [26]. In routine practice, multimodal imaging is an indispensable tool. It improves assessment of the extent and nature of posterior segment involvement, helps assess disease activity, and facilitates the monitoring of response to therapy over time [15,16,17]. Although large prospective studies are lacking, the available clinical experience and data from cohort analyses show that initial visual acuity and timely initiation of adequate antimicrobial therapy are crucial for visual recovery. This further emphasizes the importance of early etiological diagnosis in cases of posterior ocular inflammation [27,28,29,30]. Despite improved recognition of ocular syphilis, the relationship between severe posterior segment inflammation and the development of rhegmatogenous retinal detachment remains poorly understood. In practice, diagnostic algorithms that include infectious mimics are often not applied consistently, further complicating the timely establishment of an accurate diagnosis.

A particularly sensitive and often debatable issue concerns the relationship between ocular syphilis and neurosyphilis. This is particularly important when considering the indication for cerebrospinal fluid examination. Clinical observations have shown that there is a significant overlap between ocular and neurological manifestations, which has, over time, led to uncertainty about the routine use of lumbar puncture in patients with exclusively ocular symptoms [31]. Therefore, an individualized approach is increasingly advocated today. The decision to perform cerebrospinal fluid (CSF) analysis is based on the clinical presentation of syphilitic disease, the presence of neurological symptoms, the degree of diagnostic uncertainty, and risk factors such as HIV coinfection, rather than a *one-size-fits-all* approach [32].

This individualized approach is also reflected in current guidelines from the Centers for Disease Control and Prevention (CDC). Patients with ocular symptoms and reactive syphilis serology should undergo a thorough ophthalmological and neurological examination, with particular attention to cranial nerve function. CSF analysis is recommended in the presence of neurological signs but is not mandatory in isolated ocular involvement without evidence of neurological disease before initiating treatment [33]. This approach aims to avoid unnecessary invasive procedures while allowing for timely initiation of appropriate antimicrobial therapy.

Delay in diagnosis remains a major practical problem. Infectious uveitis is sometimes misinterpreted as noninfectious or viral, which may lead to premature initiation of systemic corticosteroid or immunosuppressive therapy. In such situations, the clinical condition often deteriorates before the true cause of the disease is recognized [27,28,29,30]. Although corticosteroids may have a place as adjunctive therapy, their use should only follow appropriate first-line antimicrobial therapy, especially in cases of significant posterior segment inflammation or optic nerve involvement. Expert consensus clearly warns that “corticosteroids first” strategies for unexplained posterior uveitis or panuveitis carry significant risks [9]. The available data support approaches aimed at initial investigation of all potential infectious etiology, primarily syphilis, in any unexplained posterior uveitis or panuveitis. This is especially important in cases of bilateral disease, retinal vasculitis, or optic disc edema. In such situations, early initiation of therapy for neurosyphilis should be a priority, while the decision to examine CSF should be made on an individual basis, according to clinical and diagnostic findings and in coordination between ophthalmologists and neurologists [31].

### 3.2. Retinal Detachment in Syphilitic Panuveitis

Retinal detachment (RD) in syphilitic uveitis is a rare but highly vision-threatening complication. Different phenotypes have been described in the literature, including exudative, rhegmatogenous and, less commonly, complex combined rhegmatogenous-traction forms. In most cases, RD occurs in the context of severe posterior segment inflammation and active retinitis [34]. Exudative types of retinal detachment are most commonly associated with focal inflammatory retinitis. In some cases, they may show partial or even complete resolution after timely and adequate anti-syphilitic antimicrobial therapy. In contrast, RRD in this infectious uveitis clinical setting requires vitreoretinal surgery much more often and is usually accompanied by a cautious, often unfavorable anatomical and functional prognosis [34,35,36,37,38]. In the largest contemporary synthesis dedicated to syphilitic uveitis complicated by RD, retinal detachment in a significant number of cases developed very early, shortly after the onset of uveitis. Retinal tears were most often localized in areas of previous retinitis, i.e., in zones that were already structurally weakened by the inflammatory process [36]. The pathophysiology of RD in this context is likely to be multifactorial. Severe inflammatory retinitis can lead to focal retinal necrosis, retinal thinning, and loss of mechanical stability. Such a retina is more susceptible to rupture. At the same time, persistent vitritis and inflammation-induced vitreoretinal adhesion lead to chronic retinal traction. This combination further destabilizes the vitreoretinal interface and facilitates progression to RRD [34,35,36,37]. A particularly important and often underestimated aspect is the pronounced tendency of the disease towards early onset of proliferative vitreoretinopathy (PVR). In syphilitic posterior uveitis, PVR can have a fulminant course, with intense fibroglial proliferation. This significantly increases the risk of developing complex rhegmatogenous-traction detachments and recurrent RD, even after adequate surgical treatment [34]. Bilateral RD in ocular syphilis remains extremely rare and is mostly limited to isolated case reports. When it occurs, it most often reflects an aggressive inflammatory course of the disease and/or a significant delay in establishing the diagnosis. In some situations, the use of corticosteroids before etiological confirmation of an infectious cause may also contribute to this outcome [36].

**Figure 2:**
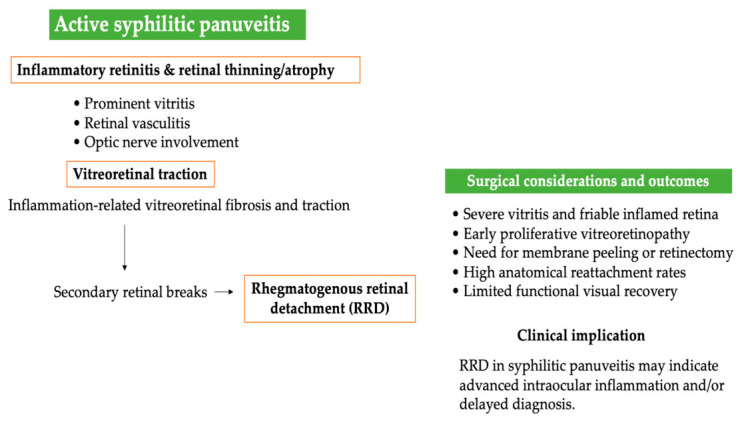
Pathophysiological and surgical continuum of retinal detachment in syphilitic panuveitis.

**Figure 3:**
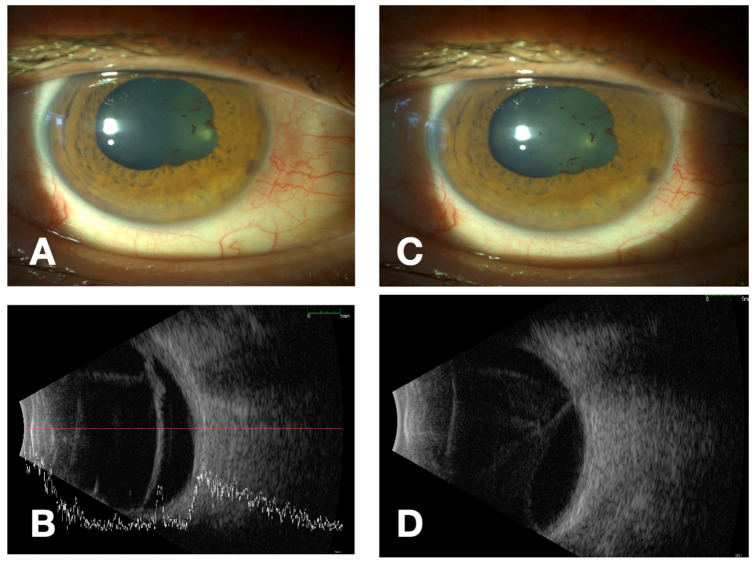
Clinical and ultrasonographic findings from our clinical practice in a patient with bilateral panuveitis complicated by uveitic cataract and combined bilateral rhegmatogenous and tractional retinal detachment (RRD). (**A**,**B**) Right eye (OD): anterior segment photograph showing marked media opacity with absent/poor red reflex, preventing fundus visualization; corresponding B-scan demonstrates dense, low-to-medium reflective vitreous echoes consistent with active vitritis, together with detached retina consistent with concomitant trational RRD. (**C**,**D**) Left eye (OS): analogous anterior segment appearance with non-viewable fundus due to media opacity; B-scan confirms diffuse vitreal inflammatory echoes (vitritis) and an associated complicated retinal detachment.

The approach to treatment of these patients is complex and requires careful timing of all therapeutic steps. The basis is the urgent initiation of antimicrobial therapy in the neurosyphilis regimen, with close monitoring and control of intraocular inflammation. Vitreoretinal surgical interventions must be carefully planned, and patients must be adequately informed about the high risk of progression of ocular disease despite treatment, PVR development and various possible postoperative complications. Even in cases of successful anatomical retinal reattachment after surgery, functional outcomes remain variable and often limited by permanent inflammatory and ischemic damage to the retina, and sometimes by the consequences of surgical treatment itself [36,37,38,39,40]. The pathophysiological continuum from active posterior segment inflammation to surgical complexity and restricted visual outcome is illustrated in Figure 2, while the key mechanisms and their clinical implications are detailed in Table 2. Ocular ultrasound represents a valuable adjunctive tool in the evaluation of severe uveitis with obscured fundus view, allowing early recognition of posterior segment pathology and helping to distinguish true inflammatory disease from infectious or masquerade syndromes when conventional imaging is limited [41]. An illustrative example of this diagnostic utility is shown in Figure 3, demonstrating characteristic ultrasonographic findings of active syphilitic involvement within the vitreous cavity and on the retina surface.

### 3.3. Diagnostic Challenges and Infectious Mimickers

Because distinct infectious entities can converge on a similar posterior segment picture, severe bilateral posterior uveitis or panuveitis with retinal vasculitis should be treated as a diagnostic red flag rather than “presumed non-infectious” inflammation. This matters most in acute or fast-evolving presentations, where starting corticosteroids or other immunosuppression before excluding infection can rapidly worsen tissue injury and visual outcome. A practical workup therefore relies on correlating the clinical presentation with multimodal imaging and targeted epidemiologic and exposure history to reach an etiologic diagnosis of posterior uveitis without delay. In parallel, clinicians should keep masquerade syndromes in mind, as both malignant and non-malignant conditions may mimic intraocular inflammation and contribute to misclassification and inappropriate early management [41].

Other clinically important mimickers of syphilitic panuveitis include tuberculosis-associated uveitis, herpetic viral retinitis, toxoplasma retinochoroiditis, and sarcoid-associated posterior uveitis. Tuberculosis may present with chronic posterior uveitis, occlusive retinal vasculitis, multifocal choroiditis, or serpiginous-like choroiditis and should be considered particularly in patients with relevant geographic, systemic, or radiologic risk factors. Herpetic viral retinitis, including HSV-, VZV-, or CMV-associated disease, may show necrotizing retinitis, retinal hemorrhages, occlusive vasculitis, and rapid progression, especially in immunocompromised individuals. Toxoplasma retinochoroiditis typically presents with focal necrotizing retinitis, often adjacent to a pigmented chorioretinal scar, accompanied by prominent vitritis. Sarcoidosis may also enter the differential diagnosis because of its association with posterior uveitis, retinal periphlebitis, choroidal lesions, and optic nerve involvement. Taken together, these entities support a broad infection-oriented and systemic diagnostic workup in patients with severe posterior segment inflammation [29,30].

Among the more context-dependent infectious mimickers, WNV-associated ocular disease predominantly affects the posterior segment and most commonly manifests as multifocal chorioretinitis, retinal vasculitis, and optic neuritis, whereas panuveitis is reported less frequently. A distinctive pattern of linear or clustered chorioretinal lesions aligned with the retinal nerve fiber layer has been consistently described and is thought to reflect viral spread along neural structures. Recognition of this imaging phenotype may provide a critical diagnostic clue in the appropriate epidemiological and seasonal context [25,42]. Given the substantial phenotypic overlap between WNV-associated ocular inflammation and ocular syphilis, particularly in bilateral cases characterized by retinal vasculitis and optic nerve involvement, clinicians practicing in endemic regions, during seasonal outbreaks, or in patients presenting with concomitant systemic or neurological features should maintain a low threshold for WNV serologic testing in serum and, when clinically indicated, cerebrospinal fluid as part of the initial diagnostic workup [14,18,40,41,42,43].

**Figure 4:**
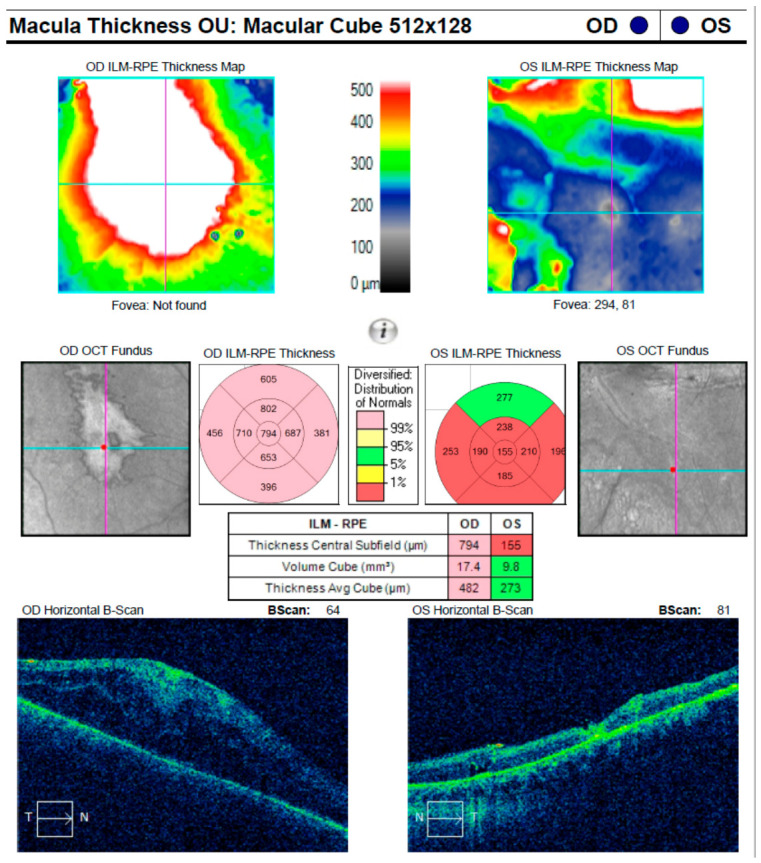
Cirrus HD-OCT macular cube scan (512 × 128) of the right eye (OD) in a patient with bilateral ocular syphilis and concomitant HIV infection, obtained postoperatively following pars plana vitrectomy (PPV) for combined rhegmatogenous and tractional retinal detachment (RRD). The image demonstrates marked macular thickening with loss of the foveal contour, consistent with severe inflammatory tractional macular edema in the right eye (central subfield thickness: 794 μm). In contrast, the left eye (OS) shows thinning of the central retinal layers (central subfield thickness: 155 μm). Color-coded thickness maps represent retinal thickness, with warmer colors (red/yellow) indicating increased thickness and cooler colors (blue) indicating thinning.

In parallel, HIV co-infection remains the most clinically consequential and well-established companion diagnosis in ocular syphilis. Shared transmission routes, combined with HIV-related immune dysregulation, are consistently associated with higher rates of bilateral disease, prominent posterior segment involvement (including retinitis, retinal vasculitis, and neuroretinitis), and an increased propensity for neurosyphilis [25,26,44]. Importantly, multiple clinical series and systematic syntheses demonstrate that ocular syphilis may serve as a sentinel manifestation of previously unrecognized HIV infection, often preceding systemic or neurological symptoms. Figure 4 presents an illustrative OCT example in a patient with bilateral ocular syphilis and concomitant HIV infection, demonstrating clinically significant macular edema with epimacular traction.

These observations underscore the necessity of routine HIV testing whenever ocular syphilis is confirmed or strongly suspected, both to guide prognosis and to ensure appropriate coordination of antimicrobial and antiretroviral therapy [45,46,47]. The most clinically relevant co-infections and infectious confounders associated with syphilitic panuveitis, together with their characteristic ocular features and diagnostic implications, are summarized in Table 3. Diagnostic delay and initial misclassification remain major drivers of poor outcomes in ocular infection associated posterior uveitis. In practice, infectious posterior uveitis is sometimes interpreted as autoimmune inflammation or presumed viral retinitis, and patients may be started on systemic corticosteroids or other immunosuppressive agents before an etiology is confirmed. This steroid-first sequence can briefly blunt intraocular inflammation while allowing the underlying pathogen-driven process to advance, widen retinal involvement, and postpone definitive antimicrobial therapy [48,49,50]. The concern is greatest in severe bilateral posterior uveitis or panuveitis with retinal vasculitis, a phenotype in which an infectious cause should be considered highly likely and actively excluded before escalating immunosuppression.

## 4. Discussion

Syphilitic panuveitis remains one of the most diagnostically demanding entities in contemporary uveitis practice, primarily due to its heterogeneous posterior segment manifestations and its tendency to mimic noninfectious or infectious inflammatory disorders [18,20,21,22]. From a clinical ophthalmology perspective, delayed etiological recognition represents the most important modifiable determinant of outcome, a finding consistently demonstrated across cohort studies and systematic analyses [21,22]. Importantly, diagnostic delay rarely reflects disease rarity, but rather phenotypic ambiguity at presentation, particularly in cases dominated by posterior segment inflammation [21,22,27].

This diagnostic uncertainty is further compounded by several infectious mimickers with overlapping clinical phenotypes. Clinically relevant differential diagnoses include tuberculosis-associated uveitis, herpetic viral retinitis, toxoplasma retinochoroiditis and, in selected settings, West Nile virus (WNV)-associated ocular inflammation. WNV should be considered primarily in the appropriate epidemiologic or seasonal context, especially when posterior uveitis or panuveitis is accompanied by retinal vasculitis, optic nerve involvement, or neurologic manifestations [14,40,41,42,46,47,48,49,50,51,52,53]. Characteristic linear or clustered chorioretinal lesions aligned with the retinal nerve fiber layer may serve as a useful distinguishing feature in such cases [14,40,41,42,46,50]. More broadly, these overlapping phenotypes support an infection-oriented diagnostic strategy in patients with unexplained severe posterior segment inflammation.

Ocular syphilis itself underscores the need for systematic consideration of treponemal infection in patients presenting with otherwise unexplained posterior segment inflammation. Multiple cohorts have shown that ocular syphilis may represent the initial manifestation of previously undiagnosed HIV infection, with coinfected patients exhibiting greater inflammatory burden and more extensive posterior segment involvement [21,22,23,24,25,26,54,55,56,57]. However, HIV status alone does not independently determine visual prognosis [26,57]. In this setting, timely diagnosis and early initiation of antiretroviral therapy contribute to systemic stabilization and improved control of intraocular inflammation [21,57].

A particularly important clinical issue highlighted in several series is the premature use of corticosteroids before etiological confirmation. Although transient inflammatory suppression may occur, early corticosteroid exposure in untreated syphilitic uveitis has been associated with delayed diagnosis, persistent pathogen-driven disease activity, and worse anatomic and visual outcomes [21,22,27,44,45]. Consistent with prognostic analyses, early clinical response following initiation of appropriate antimicrobial therapy appears to be a stronger predictor of outcome than initial disease severity or specific antimicrobial regimen [21,22]. Conversely, escalation of periocular or systemic corticosteroid therapy before adequate antimicrobial coverage may adversely affect visual recovery, emphasizing the importance of careful sequencing of anti-inflammatory treatment.

### 4.1. Ophthalmo-Surgical Considerations

**Figure 5:**
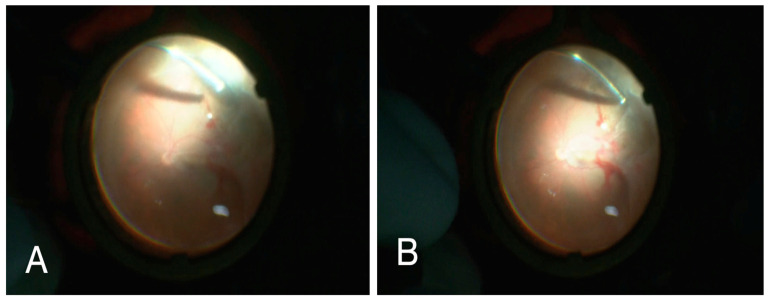
Representative intraoperative wide-angle view during pars plana vitrectomy in a patient with panuveitis complicated by bilateral rhegmatogenous retinal detachment. (**A**,**B**) Media haze from inflammatory vitreous debris limits initial intraoperative fundus visualization, and the retina is complexly detached with prominent traction, fragility, extensive subretinal fluid and poor retinal stability. This illustrates key surgical challenges—restricted visualization, inflamed fragile and necrotic tissues, and difficult controlled reattachment due to traction.

Rhegmatogenous retinal detachment (RRD) represents a rare but particularly severe complication of syphilitic uveitis and should be regarded as a structural marker of advanced inflammation-induced vitreoretinal instability rather than an isolated event. Even after apparent control of active inflammation, persistent vitreoretinal traction and early proliferative vitreoretinopathy may limit both anatomical and functional outcomes, emphasizing the importance of early etiological diagnosis and close posterior segment monitoring [34,35,36,37,38,39,40]. From a surgical standpoint, these cases remain among the most challenging scenarios in vitreoretinal surgical practice, with prognosis largely determined by the extent of preexisting complex inflammatory damage rather than intraoperative success alone [37,38,39,40]. Pars plana vitrectomy is the main surgical approach in eyes with rhegmatogenous retinal detachment occurring in the setting of syphilitic panuveitis, particularly in the presence of dense vitritis, vitreoretinal traction, limited fundus visualization, or combined rhegmatogenous-tractional components. The choice of intraocular tamponade should be individualized according to the extent and configuration of detachment, location of retinal breaks, degree of inflammatory activity, and anticipated risk of postoperative proliferative vitreoretinopathy. In more complex inflammatory detachments, silicone oil may provide more prolonged internal support and facilitate postoperative retinal stabilization, whereas expansile gas tamponade may be appropriate in selected, less complex cases. However, disease-specific the comparative evidence regarding tamponade selection in syphilitic uveitis remains limited. Intraoperative findings in our representative case of bilateral syphilitic uveitis underscore this point, demonstrating severe inflammatory vitreoretinal pathology and complex retinal detachment configuration (Figure 5). Taken together, these observations support a diagnostic and therapeutic strategy centered on early etiological recognition, infection-oriented evaluation, and careful sequencing of antimicrobial and local and systemic anti-inflammatory therapy. In syphilitic panuveitis, outcomes are shaped less by the intensity of initial inflammation and more by the timeliness and accuracy of diagnosis and the early trajectory of clinical response, a principle that should guide both medical and ophthalmo-surgical decision-making in daily practice [21,22]. Silicone oil tamponade may also contribute to transient postoperative changes in central macular thickness parameters, most of which tend to normalize after oil removal, although mechanisms remain uncertain and may warrant correlation with choroidal thickness, the peripapillary retinal nerve fiber layer, and choriocapillaris perfusion changes [58,59]. Moreover, uveitic cataract can significantly complicate the intraoperative course by limiting visualization and increasing surgical complexity [60].

### 4.2. Strength of Evidence and Evidence Gaps

The available evidence on syphilitic panuveitis and associated rhegmatogenous retinal detachment remains limited. Most of the published literature consists of retrospective cohort studies, small case series, and selected case reports, with considerable heterogeneity in patient characteristics, HIV status, imaging modalities, treatment approaches, and outcome reporting. Accordingly, several clinically important conclusions, particularly those concerning the mechanisms, timing, and prognosis of rhegmatogenous retinal detachment, are supported by a low to moderate strength of evidence rather than by robust comparative studies. Major evidence gaps include the lack of prospective investigations, limited standardization in the reporting of vitreoretinal complications and surgical outcomes, and insufficient data on how infectious mimickers are incorporated into real world diagnostic algorithms. These limitations should be taken into account when interpreting the current literature and applying its findings in clinical practice.

### 4.3. Clinical Implications for Practice

1. Syphilitic panuveitis should be routinely considered in all patients presenting with unexplained posterior uveitis or panuveitis, irrespective of perceived demographic or behavioral risk factors. Reliance on presumed risk profiles may delay recognition of this diagnosis and contribute to avoidable visual morbidity.
2. Early serologic testing for syphilis and HIV is essential, particularly in cases of bilateral disease, retinal vasculitis, or optic disc edema, as these features frequently reflect an underlying infectious etiology and have direct implications for treatment strategy and prognosis.
3. The presence of rhegmatogenous retinal detachment should be regarded as a marker of advanced disease, prompting close posterior segment monitoring and early involvement of a vitreoretinal surgeon, given the high risk of rapid disease progression and the consequent surgical complexity.
4. Corticosteroid-first treatment strategies should be avoided until infectious causes have been reasonably excluded or appropriate antimicrobial therapy has been initiated, as premature immunosuppression may exacerbate infection, promote ocular tissue damage and worsen visual outcomes.
5. Targeted evaluation for infectious mimickers, including WNV in appropriate epidemiological or seasonal contexts, should be incorporated into the diagnostic algorithm to minimize diagnostic delay and reduce the risk of irreversible vision loss.

### 4.4. Limitations

This review has several limitations. First, its narrative design does not offer the methodological rigor of a formal systematic review or meta-analysis. Second, the available evidence is derived predominantly from retrospective studies, small case series, and selected case reports, which limits the strength, consistency, and generalizability of the conclusions. Third, publication bias is likely, particularly in relation to rare and surgically complex presentations such as rhegmatogenous retinal detachment, which are more frequently presented in the published literature. Furthermore, substantial heterogeneity in clinical phenotypes, imaging findings, treatment strategies, and outcome reporting precluded quantitative synthesis. Accordingly, this review should be interpreted as a clinically oriented integrative overview rather than as a definitive estimate of incidence, prognosis, or treatment effect.

## 5. Conclusions

Syphilitic panuveitis represents one of the most challenging entities in contemporary uveitis practice because of its broad clinical spectrum, frequent posterior-segment involvement, and potential to cause permanent structural and functional visual impairment. RRD, although rare, should be regarded as a marker of advanced inflammation-induced vitreoretinal instability. It is often associated with surgical complexity, an increased risk of proliferative vitreoretinopathy, and uncertain anatomical and functional outcomes. Ophthalmologists should initiate serological testing for syphilis without delay in patients with unexplained posterior uveitis or panuveitis, particularly in the presence of bilateral disease, retinal vasculitis, or optic disc involvement. Once the diagnosis is suspected or confirmed, neurosyphilis-directed antimicrobial therapy should be initiated promptly, with careful planning of adjunctive anti-inflammatory treatment.

Finally, given the frequent association of ocular syphilis with HIV coinfection and the existence of relevant infectious mimickers, targeted diagnostic evaluation should be incorporated early into the clinical algorithm. Effective management of syphilitic panuveitis depends on early diagnosis and coordinated multidisciplinary care, particularly between ophthalmology, infectious disease, and neurology, in order to reduce delays and prevent avoidable visual loss.

## Figures and Tables

**Table 1:** Key contemporary studies on ocular syphilis presenting as posterior uveitis/panuveitis: study characteristics and selected quantitative findings.

Author (Year)	Design	Population	Presentation	Selected Quantitative Findings	Key Points
Guideline (CDC STI Treatment Guidelines) (2021) [19]	Clinical practice guideline	Patients with syphilis and ocular involvement	Posterior uveitis, panuveitis	Not applicable	Ocular syphilis should be treated using neurosyphilis regimens; CSF evaluation guided by neurologic findings
Dutta Majumder et al. (2019) [21]	Narrative review	Mixed ocular syphilis cohorts	Posterior uveitis, panuveitis	Quantitative synthesis limited by heterogeneity	Highlights frequent diagnostic delay, misclassification, and need for early etiologic therapy
Furtado et al. (2022) [22]	Comprehensive review	Ocular syphilis across clinical settings	Posterior uveitis, panuveitis	Quantitative comparison limited across studies	Emphasizes posterior segment-predominant disease and the role of multimodal imaging
Mathew & Smit (2021) [23]	Retrospective cohort study	Patients with ocular and neurosyphilis ± HIV	Posterior uveitis, panuveitis	HIV coinfection: 52.1%; posterior uveitis: 40.9%; panuveitis: 38.1%	HIV co-infection associated with increased severity and posterior segment involvement
Kunkel et al. (2009) [24]	Observational study	Patients with ocular syphilis	Posterior uveitis, panuveitis	Ocular syphilis was reported as a possible presenting manifestation of previously undiagnosed HIV infection	Ocular involvement may be the first clinical manifestation of undiagnosed HIV
Bollemeijer et al. (2016) [6]	Retrospective cohort study	Patients with syphilitic uveitis	Posterior uveitis, panuveitis	HIV coinfection: 35.9%; posterior uveitis: 31.8%; panuveitis: 45.9%; 86.7% achieved remission	Demonstrates frequent posterior segment disease and generally favorable response with timely treatment
Pichi et al. (2020) [15]	Literature review	Posterior syphilitic uveitis	Posterior uveitis	Quantitative synthesis not primary aim	Defines characteristic multimodal imaging findings (FA, OCT, UWF)
Eandi et al. (2012) [7]	Case series + literature review	Syphilitic uveitis	Posterior uveitis predominance	Quantitative data limited by small sample size	Supports multimodal imaging for diagnosis and disease monitoring
Tucker et al. (2011) [25]	Systematic analysis of the literature	HIV-infected patients with ocular syphilis	Predominantly posterior segment involvement	52% newly diagnosed with HIV at ocular presentation; 97% of visually impaired patients improved after treatment	Ocular syphilis may be a sentinel manifestation of previously unrecognized HIV infection

Abbreviations: CDC, Centers for Disease Control and Prevention; STI, sexually transmitted infection; CSF, cerebrospinal fluid; FA, fluorescein angiography; OCT, optical coherence tomography; UWF, ultra-widefield imaging.

**Table 2:** Retinal detachment associated with syphilitic panuveitis: proposed mechanisms and clinical implications.

Aspect	Key Features	Clinical Relevance
Type of retinal detachment	Exudative and rhegmatogenous retinal detachment; combined rhegmatogenous–tractional forms in the setting of severe inflammation	The type of detachment directly influences urgency and management. Exudative detachment may improve with appropriate antimicrobial therapy, whereas rhegmatogenous detachment usually requires surgical intervention and is associated with a guarded prognosis [26]
Retinal structural damage	Inflammation-associated retinitis with retinal thinning and focal necrosis; retinal breaks often develop in areas of previous retinitis	Structural weakening of the retina increases susceptibility to tears and facilitates progression toward rhegmatogenous retinal detachment [10,12]
Vitreoretinal interface	Persistent vitritis with inflammation-driven vitreoretinal adhesion and traction	Vitreoretinal traction contributes to retinal break formation, promotes rhegmatogenous detachment, and increases surgical complexity [34,35]
Proliferative vitreoretinopathy (PVR)	Early onset and often aggressive fibroglial proliferation	One of the main determinants of retinal redetachment risk and poor functional visual outcome [38,39,40]
Bilaterality and diagnostic delay	Bilateral involvement is rare and mainly described in isolated case reports; frequently associated with delayed diagnosis or prior corticosteroid exposure	Suggests a more aggressive disease course and highlights the importance of early etiologic evaluation before initiating immunosuppressive therapy [26,29]
Visual and anatomical outcome	Anatomical reattachment can often be achieved; functional recovery remains unpredictable	Visual outcomes are frequently limited by irreversible inflammatory and ischemic retinal damage and postoperative complications [10,39]

**Table 3:** Clinically relevant co-infections and mimickers of syphilitic panuveitis and their diagnostic implications.

Co-Infection/Mimic	Typical Ocular Manifestations	Clinical/Diagnostic Implications
Tuberculosis-associated uveitis	Chronic posterior uveitis; occlusive retinal vasculitis; multifocal choroiditis; serpiginous-like choroiditis	Important differential diagnosis in patients with compatible epidemiologic exposure, systemic findings, or chest imaging abnormalities. Supports targeted tuberculosis-oriented evaluation in selected cases of severe posterior uveitis or panuveitis [13,28].
Herpetic viral retinitis (HSV, VZV, CMV)	Necrotizing retinitis; retinal hemorrhages; occlusive retinal vasculitis; dense vitritis; rapidly progressive posterior segment inflammation	Important differential diagnosis, particularly in immunocompromised patients. Early recognition is essential because delayed antiviral treatment may lead to rapid retinal destruction and severe visual loss [13,28].
Toxoplasma retinochoroiditis	Focal necrotizing retinitis/retinochoroiditis, often adjacent to a pigmented chorioretinal scar; prominent vitritis	May resemble infectious posterior uveitis and should be considered particularly when focal retinitis with dense vitritis is present. Careful fundus examination and correlation with clinical context are essential [13,28].
Sarcoid-associated posterior uveitis	Posterior uveitis; retinal periphlebitis; choroidal lesions; optic nerve involvement	May mimic ocular syphilis because of overlapping posterior inflammatory findings. Supports broader systemic differential diagnosis when infectious testing is inconclusive or when multisystem inflammatory features are present [13,28].
West Nile virus (WNV)	Multifocal chorioretinitis, retinal vasculitis, optic neuritis; panuveitis less commonly observed; characteristic linear or clustered lesions distributed along the retinal nerve fiber layer	May mimic ocular syphilis, particularly in endemic regions or during seasonal outbreaks. Targeted WNV serologic testing should be considered when epidemiologic context or accompanying systemic or neurologic features raise suspicion [14,40,41,42,46,47,48,49,50,51,52,53].
HIV	Posterior uveitis or panuveitis; retinitis or chorioretinitis; retinal vasculitis; optic nerve involvement, including neuroretinitis and optic disc edema	Frequently associated with bilateral disease, predominant posterior segment involvement, and an increased likelihood of neurosyphilis. Routine HIV testing is recommended once ocular syphilis is suspected or confirmed [23,24,25,26,54,55,56,57].
Autoimmune misclassification	Apparent early responsiveness to corticosteroids; posterior uveitis or panuveitis resembling non-infectious inflammation	Premature corticosteroid or immunosuppressive therapy may worsen untreated infection and prolong diagnostic delay. Emphasizes the need for early syphilis testing and avoidance of corticosteroid-first strategies [27,44].

## Data Availability

No new data were created or analyzed in this study.

## Abbreviations

The following abbreviations are used in this manuscript:

CSF	Cerebrospinal fluid
CDC	Centers for Disease Control and Prevention
HIV	Human immunodeficiency virus
HSV	Herpes simplex virus
PVR	Proliferative vitreoretinopathy
RD	Retinal detachment
PPV	Pars plana vitrectomy
RRD	Rhegmatogenous retinal detachment
UWF	Ultra-widefield imaging
VZV	Varicella-zoster virus
WNV	West Nile virus
